# A simplified approach to discriminate between healthy subjects and patients with heart failure using cardiac magnetic resonance myocardial deformation imaging

**DOI:** 10.1093/ehjimp/qyae093

**Published:** 2024-09-12

**Authors:** Undine Ella Witt, Maximilian Leo Müller, Rebecca Elisabeth Beyer, Johannes Wieditz, Susanna Salem, Djawid Hashemi, Wensu Chen, Mina Cvetkovic, Anna Clara Nolden, Patrick Doeblin, Moritz Blum, Gisela Thiede, Alexander Huppertz, Henning Steen, Bjoern Andrew Remppis, Volkmar Falk, Tim Friede, Sebastian Kelle

**Affiliations:** Department of Cardiology, Deutsches Herzzentrum der Charité, Angiology and Intensive Care Medicine, Augustenburger Platz 1, Berlin 13353, Germany; Corporate Member of Freie Universität Berlin and Humboldt-Universität zu Berlin, Charité—Universitätsmedizin Berlin, Berlin, Germany; Herzinstitut Berlin, Kardiologische Gemeinschaftspraxis, Berlin, Germany; Department of Cardiology, Deutsches Herzzentrum der Charité, Angiology and Intensive Care Medicine, Augustenburger Platz 1, Berlin 13353, Germany; Corporate Member of Freie Universität Berlin and Humboldt-Universität zu Berlin, Charité—Universitätsmedizin Berlin, Berlin, Germany; DZHK (German Centre for Cardiovascular Research), Partner Site Berlin, Berlin, Germany; Department of Cardiology, Deutsches Herzzentrum der Charité, Angiology and Intensive Care Medicine, Augustenburger Platz 1, Berlin 13353, Germany; Corporate Member of Freie Universität Berlin and Humboldt-Universität zu Berlin, Charité—Universitätsmedizin Berlin, Berlin, Germany; DZHK (German Centre for Cardiovascular Research), Partner Site Berlin, Berlin, Germany; Department of Medical Statistics, University Medical Center Göttingen, Göttingen, Germany; Department of Medical Statistics, University Medical Center Göttingen, Göttingen, Germany; Department of Cardiology, Deutsches Herzzentrum der Charité, Angiology and Intensive Care Medicine, Augustenburger Platz 1, Berlin 13353, Germany; Corporate Member of Freie Universität Berlin and Humboldt-Universität zu Berlin, Charité—Universitätsmedizin Berlin, Berlin, Germany; DZHK (German Centre for Cardiovascular Research), Partner Site Berlin, Berlin, Germany; DZHK (German Centre for Cardiovascular Research), Partner Site Berlin, Berlin, Germany; Department of Cardiology, Affiliated Hospital of Xuzhou Medical University, Xuzhou, China; Department of Cardiology, Deutsches Herzzentrum der Charité, Angiology and Intensive Care Medicine, Augustenburger Platz 1, Berlin 13353, Germany; Corporate Member of Freie Universität Berlin and Humboldt-Universität zu Berlin, Charité—Universitätsmedizin Berlin, Berlin, Germany; Department of Cardiology, Deutsches Herzzentrum der Charité, Angiology and Intensive Care Medicine, Augustenburger Platz 1, Berlin 13353, Germany; Corporate Member of Freie Universität Berlin and Humboldt-Universität zu Berlin, Charité—Universitätsmedizin Berlin, Berlin, Germany; DZHK (German Centre for Cardiovascular Research), Partner Site Berlin, Berlin, Germany; Department of Cardiology, Deutsches Herzzentrum der Charité, Angiology and Intensive Care Medicine, Augustenburger Platz 1, Berlin 13353, Germany; Corporate Member of Freie Universität Berlin and Humboldt-Universität zu Berlin, Charité—Universitätsmedizin Berlin, Berlin, Germany; DZHK (German Centre for Cardiovascular Research), Partner Site Berlin, Berlin, Germany; Corporate Member of Freie Universität Berlin and Humboldt-Universität zu Berlin, Charité—Universitätsmedizin Berlin, Berlin, Germany; Department of Cardiothoracic and Vascular Surgery, Deutsches Herzzentrum der Charité, Augustenburger Platz 1, Berlin 13353, Germany; BIH Biomedical Innovation Academy, BIH Charité Junior Clinician Scientist Program, Berlin Institute of Health at Charité—Universitätsmedizin Berlin, Charitéplatz 1, Berlin 10117, Germany; Department of Cardiology, Deutsches Herzzentrum der Charité, Angiology and Intensive Care Medicine, Augustenburger Platz 1, Berlin 13353, Germany; Corporate Member of Freie Universität Berlin and Humboldt-Universität zu Berlin, Charité—Universitätsmedizin Berlin, Berlin, Germany; DZHK (German Centre for Cardiovascular Research), Partner Site Berlin, Berlin, Germany; University Outpatient Clinic, Sports Medicine and Sports Orthopaedics, University of Potsdam, Potsdam, Germany; Department of Cardiology, Angiology and Pneumology, Heidelberg University, Heidelberg, Germany; Department of Cardiology, Cardiovascular Center Bad Bevensen, Bad Bevensen, Germany; Corporate Member of Freie Universität Berlin and Humboldt-Universität zu Berlin, Charité—Universitätsmedizin Berlin, Berlin, Germany; Department of Cardiothoracic and Vascular Surgery, Deutsches Herzzentrum der Charité, Augustenburger Platz 1, Berlin 13353, Germany; Department of Health Sciences and Technology, Institute of Translational Medicine, Translational Cardiovascular Technologies, Swiss Federal Institute of Technology (ETH) Zurich, Zurich, Switzerland; DZHK (German Centre for Cardiovascular Research), Partner Site Berlin, Berlin, Germany; Department of Cardiology, Deutsches Herzzentrum der Charité, Angiology and Intensive Care Medicine, Augustenburger Platz 1, Berlin 13353, Germany; Corporate Member of Freie Universität Berlin and Humboldt-Universität zu Berlin, Charité—Universitätsmedizin Berlin, Berlin, Germany; DZHK (German Centre for Cardiovascular Research), Partner Site Berlin, Berlin, Germany

**Keywords:** global longitudinal strain, cut-off, early identification of heart failure, cardiac magnetic resonance imaging, deformation imaging

## Abstract

**Aims:**

Left ventricular global longitudinal strain (LV-GLS) shows promise as a marker to detect early heart failure (HF). This study sought to (i) establish cardiac magnetic resonance imaging (CMR)–derived LV-GLS cut-offs to differentiate healthy from HF for both acquisition-based and post-processing techniques, (ii) assess agreement, and (iii) provide a method to convert LV-GLS between both techniques.

**Methods and results:**

A secondary analysis of a prospective study enrolling healthy subjects (*n* = 19) and HF patients (*n* = 56) was conducted. LV-GLS was measured using fast strain–encoded imaging (fSENC) and feature tracking (FT). Receiver operating characteristic (ROC) analyses were performed to derive and evaluate LV-GLS cut-offs discriminating between healthy, HF with mild deformation impairment (DI), and HF with severe DI. Linear regression and Bland–Altman analyses assessed agreement. Cut-offs discriminating between healthy and HF were identified at −19.3% and −15.1% for fSENC and FT, respectively. Cut-offs of −15.8% (fSENC) and −10.8% (FT) further distinguished mild from severe DI. No significant differences in area under ROC curve were identified between fSENC and FT. Bland–Altman analysis revealed a bias of −4.01%, 95% CI −4.42, −3.50 for FT, considering fSENC as reference. Linear regression suggested a factor of 0.76 to rescale fSENC-derived LV-GLS to FT. Using this factor on fSENC-derived cut-offs yielded rescaled FT LV-GLS cut-offs of −14.7% (healthy vs. HF) and −12% (mild vs. severe DI).

**Conclusion:**

LV-GLS distinguishes healthy from HF with high accuracy. Each measurement technique requires distinct cut-offs, but rescaling factors facilitate conversion. An FT-based LV-GLS ≥ −15% simplifies HF detection in clinical routine.

## Introduction

Heart failure (HF) is a major global public health problem, already affecting an estimated 64 million people and accounting for more than US$300 billion in healthcare expenditure worldwide.^1^ Yet, experts fear that the prevalence of HF will continue to rise, mainly due to societal ageing and increasingly unhealthy lifestyles.^2^ Therefore, detection and intervention at early pathophysiological stages are crucial to prevent the development of manifest HF.

Risk factors and the mechanisms by which they promote the development of HF are well characterized. Co-morbidities, such as hypertension, diabetes mellitus, or coronary artery disease, induce subtle but progressive myocardial damage, which eventually leads to an increased risk of transitioning to manifest HF.^3,4^ Although effective in diagnosing and monitoring symptomatic HF, traditional imaging parameters, such as the left ventricular ejection fraction (LVEF), fail to detect these subclinical changes in myocardial structure and function, rendering an identification of patients in early disease stages difficult.^5^

Deformation imaging using global and regional myocardial strain quantification has emerged as a more detailed and robust method to assess myocardial contractility.^6–8^ Indeed, mounting evidence suggests that LV global longitudinal strain (LV-GLS) is superior to LVEF in identifying subclinical myocardial changes in patients at high risk of developing overt HF.^9–11^

Several methods to quantify myocardial strain using cardiac magnetic resonance imaging (CMR) have been proposed.^12–14^ Of these, conventional myocardial tagging is the most extensively validated, but its clinical utility suffers from low spatial resolution and long acquisition and post-processing times.^12–14^ These drawbacks have been partially overcome by the introduction of fast strain–encoded imaging (fSENC), which features reduced acquisition and post-processing times.^12,13^ Yet, fSENC still requires the acquisition of dedicated sequences.^12,13^ By contrast, myocardial feature tracking (FT) facilitates the quantification of strain indices using routinely acquired cine CMR images, allowing strain measures even on a retrospective basis, when the CMR has been already completed.^12,13^

As a result, FT is now the method of choice for many clinicians.^12,13^ However, myocardial strain values differ significantly between methods, and data from previous studies suggest that acquisition-based techniques may be superior to FT in detecting myocardial dysfunction.^12,14–16^

The aim of this study was to evaluate acquisition-based (i.e. fSENC) and post-processing (i.e. FT) techniques for the identification of myocardial dysfunction in a well-characterized cohort of HF patients. Specifically, we sought to investigate LV-GLS cut-offs for the simplified identification of patients with HF, to examine the correlation of LV-GLS measurements using both techniques, and to provide a basis for the conversion of LV-GLS values derived using one technique to the other.

## Methods

### Study population

This is a secondary analysis of a prospective study conducted at Charité—Universitätsmedizin Berlin and the German Heart Centre Berlin between 2017 and 2018 (EMPATHY-HF), which enrolled healthy volunteers and subjects with HF.^11,17–24^ Subjects with HF were stratified into HF with preserved EF (HFpEF; LVEF ≥ 50% and increased NT-proBNP), HF with mildly reduced EF (HFmrEF; LVEF = 40–49% and increased NT-proBNP), and HF with reduced EF (HFrEF; LVEF < 40%) according to the diagnostic criteria outlined in the 2016 ESC HF guidelines.^25^ The study was approved by the local ethics committee and registered with the German Clinical Trials Registry (Registration ID: DRKS00015615).^24^ All patients provided written informed consent for their participation, and the study was conducted in accordance with the Declaration of Helsinki. Various other analyses utilizing the data gathered in this study have previously been published.^11,17–23^ Detailed study information, including the rationale, inclusion and exclusion criteria for all subgroups, and primary and secondary endpoints, can be found in previous publications and on the German Clinical Trials Registry website.^11,17–24^

### CMR and image analysis

CMR examinations were performed in a supine position on a whole-body 1.5 T Philips Achieva system (Philips Healthcare, Best, The Netherlands) equipped with a five-element cardiac surface coil, as described in previous analyses of this study.^11,17–24^ In brief, an initial survey was used to define the imaging planes. Cine images, including a short-axis stack, as well as two- (2CH), three- (3CH), and four-chamber (4CH) long-axis views, were acquired using a retrospectively gated breath hold steady-state free precession (bSSFP) sequence. These were used for volumetry and FT strain analyses. Additionally, 2CH, 3CH, 4CH, and three short-axis images (basal, mid-ventricular, and apical each) were acquired using a real-time free-breathing fSENC technique (Myocardial Solutions Version, Inc., Morrisville, NC, USA), technical details of which have been described previously, to facilitate fSENC strain analyses.^26^

All acquired images were analysed offline and in accordance with current consensus recommendations for the standardized image interpretation and post-processing in CMR.^27^ LV-GLS was assessed using fSENC and FT for each subject. Specifically, FT strain analyses were performed using Circle Cardiovascular Imaging 42 version 5.13.5 (CVI42^®^, Circle Cardiovascular Imaging Inc., Calgary, Canada), and fSENC strain analyses were performed using MyoStrain^®^ version 5.0 (Myocardial Solutions, Inc., Morrisville, NC, USA). Inter-study, inter-observer, and intra-observer reproducibility of fSENC- and FT-based LV-GLS measurements have previously been demonstrated by our group in this cohort.^12,28^

### Statistical analysis

For statistical analyses, study subjects were divided into healthy volunteers, HFpEF, HFmrEF, and HFrEF subgroups, as outlined above. Statistics were performed for the entire cohort and the respective healthy volunteers, HFpEF, HFmrEF, and HFrEF subgroups, using R version 4.3.3 (The R Foundation for Statistical Computing, Vienna, Austria). For the computation and evaluation of receiver operating characteristic (ROC) curves, the package pROC was employed.^29^

ROC analyses were performed to derive LV-GLS cut-offs facilitating a re-stratification of the study subjects into the following new groups: healthy subjects; HF with mild deformation impairment (DI) (i.e. HFpEF and HFmrEF); and HF with severe DI (i.e. HFrEF). Cut-offs were chosen to achieve a sensitivity of ≥85% at maximum specificity for both measured LV-GLS quantification methods (i.e. fSENC and FT). The diagnostic performance of different LV-GLS cut-offs was assessed by calculating sensitivity, specificity, negative and positive predictive values, and accuracy. Confidence intervals (CIs) of performance measures are based on 2000 bootstrap replicates. ROC curves were compared using DeLong’s test.^30^ For comparisons of area under ROC curves (AUROCs) against an uninformative ROC (random classifier), a Wilcoxon test was employed.^31^

Moreover, the agreement between FT- and fSENC-derived LV-GLS values was assessed using Bland–Altman analyses. Limits of agreement are determined at the 95% agreement level, i.e. bias ± 1.96 SD of the differences. Associations between the two methods, as well as differences of methods and given mean LV-GLS values, were analysed using linear regression models. fSENC-derived LV-GLS values were considered as the reference for these analyses. For both quantification methods, pairwise comparisons of LV-GLS values were performed between HF entities (i.e. healthy, HFpEF, HFmrEF, and HFpEF). If not stated otherwise, tests were performed two-sided at the 5% significance level.

## Results

### Patient characteristics

A total of 75 participants, including 19 healthy volunteers, as well as 19 HFpEF, 19 HFmrEF, and 18 HFrEF patients, were included in this analysis. Baseline characteristics of all patients, including the healthy volunteers, HFpEF, HFmrEF, and HFrEF subgroups, have been comprehensively summarized in previously published analyses of this cohort.^11,17–24^

### Derivation of LV-GLS cut-offs

**Figure 1:**
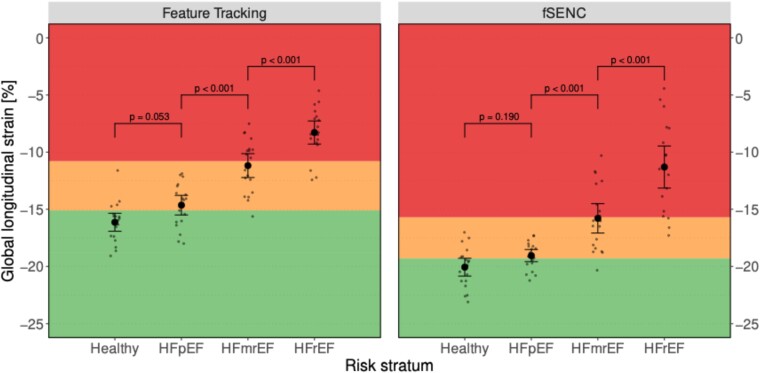
Scatter plot for fSENC- and FT-derived LV-GLS, stratified by entity of HF. Within-group means are shown as point with corresponding 95% CI (error bar). Individual measurements are shown as small dots (jittered horizontally for better visibility). Proposed cut-offs border the coloured regions. Specifically, LV-GLS cut-offs to discriminate between healthy subjects (green area) and those with HF (yellow and red area) were set at −19.3% for fSENC and at −15.1% for feature tracking. Cut-offs to distinguish between HF with mild (yellow area) and severe DI (red area) were set at −15.7% for fSENC and at −10.8% for feature tracking. *P*-values of tests for differences in mean are given above brackets indicating which HF entities are compared.

fSENC- and FT-derived LV-GLS values across different entities of HF are summarized in *Table 1*. Significant differences in mean LV-GLS were observed for both fSENC and FT when comparing HFpEF vs. HFmrEF and HFmrEF vs. HFrEF (all *P* < 0.001). In contrast, no significant differences were observed when comparing healthy vs. HFpEF using either fSENC- (*P* = 0.190) or FT-derived LV-GLS (*P* = 0.053) (*Figure 1*).

**Table 1:** Summary of LV-GLS values derived through fSENC and FT across the study subgroups

		fSENC LV-GLS				FT LV-GLS			
Subgroup	*n*	Mean (%)	SD	Lower 95% CI	Upper 95% CI	Mean (%)	SD	Lower 95% CI	Upper 95% CI
HFrEF	18	−11.3	3.98	−13.3	−9.3	−8.28	2.19	−9.4	−7.2
HFmrEF	19	−15.8	2.86	−17.2	−14.4	−11.2	2.32	−12.3	−10.1
HFpEF	19	−19.1	1.18	−19.6	−18.5	−14.6	1.91	−15.6	−13.7
Healthy	19	−20.1	1.73	−20.9	−19.2	−16.1	1.75	−17.0	−15.3

LV-GLS values (in %) are presented as mean with standard deviation and corresponding 95% CI.

**Figure 2:**
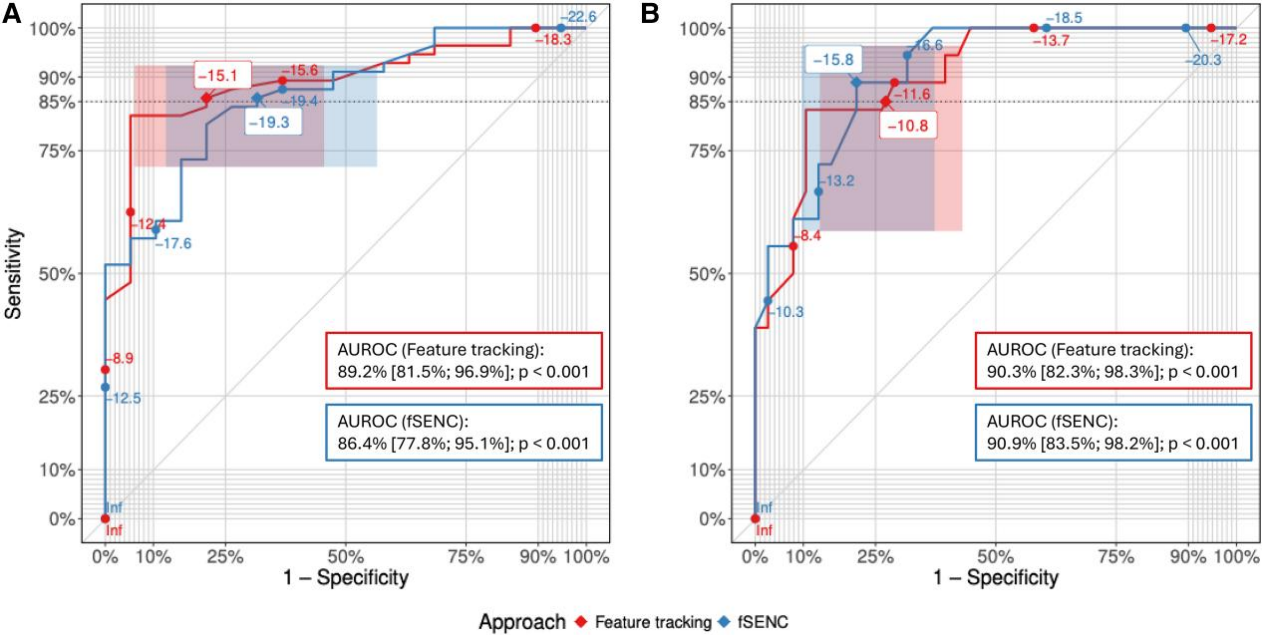
ROC curves of the comparisons of FT-based (red) and fSENC-based (blue) LV-GLS for the discrimination between (*A*, left) healthy subjects and HF patients and (*B*, right) HF with mild DI and HF with severe DI. Proposed LV-GLS cut-off values have been labelled with white boxes and positions on the ROC curve are marked with diamonds (◆). Corresponding CIs for sensitivity and specificity are represented as shaded boxes. Targeted minimum sensitivity (i.e. 85%) is indicated as a dotted horizontal line. AUROCs for the discrimination between (*A*, left) healthy subjects and patients with HF and (*B*, right) HF with mild DI and HF with severe DI are reported for fSENC and FT. *P*-values correspond to a test of the AUROC being equal to 50% (random classification).

ROC analyses, the results of which are summarized in *Figure 2*, were performed to derive cut-offs discriminating between healthy subjects and patients with HF, as well as between HF with mild DI (i.e. HFpEF and HFmrEF) and severe DI (i.e. HFrEF). Accordingly, cut-offs for fSENC-derived LV-GLS were set at −19.3% and −15.8% to distinguish healthy vs. HF and HF with mild DI vs. HF with severe DI. Corresponding cut-off levels for FT-derived LV-GLS were identified at −15.1% and −10.8% (*Table 2*).

**Table 2:** Summary of performance measures of the fSENC-derived, FT-derived, and rescaled FT LV-GLS cut-offs for the discrimination between healthy subjects and patients with HF, as well as between HF with mild DI and HF with severe DI

	Healthy vs. HF			HF with mild DI vs. HF with severe DI		
Performance parameter	fSENC	FT	rescaled FT	fSENC	FT	rescaled FT
Threshold	−19.3%	−15.1%	−14.7%	−15.8%	−10.8%	−12%
Sensitivity	85.7% (76.8%; 94.6%)	85.7% (76.7%; 94.6%)	83.9% (75%; 92.9%)	88.9% (72.2%; 100%)	85% (66.7%; 100%)	88.9% (72.2%; 100%)
Specificity	68.4% (47.4%; 89.5%)	78.9% (57.9%; 94.7%)	79% (57.9%; 94.7%)	79% (65.8%; 92.1%)	73.7% (57.9%; 86.8%)	65.8% (50%; 79%)
Positive predictive value	88.9% (82.4%; 95.7%)	92.5% (86.8%; 98%)	92.5% (85.7%; 98%)	66.7% (53.9%; 82.4%)	60.7% (46.7%; 76.2%)	55.2% (45%; 68.2%)
Negative predictive value	61.9% (45.8%; 80%)	65.4% (51.6%; 82.6%)	62.5% (48.3%; 79%)	93.9% (85.7%; 100%)	90.6% (80.8%; 100%)	92.9% (82.8%, 100%)
Accuracy	81.3% (72%; 89.3%)	84% (76%; 92%)	82.7% (73.3%; 90.7%)	82.1% (71.4%; 91.1%)	76.8% (64.3%; 87.5%)	73.2% (62.5%; 83.9%)
AUROC	86.4% (77.8%; 95.1%)^a^	89.2% (81.5%; 96.9%)^a^		90.9% (83.5%; 98.2%)^a^	90.3% (82.3%; 98.3%)^a^	
Comparison between AUROC of fSENC and FT	*P* = 0.406			*P* = 0.831		

AUROCs regarding the differentiation between healthy subjects and patients with HF, as well as between HF with mild DI and HF with severe DI, are reported for continuous fSENC- and FT-derived GLS with ‘a’ indicating a significantly different AUROC compared with a random classifier (*P* < 0.001). Additionally, *P*-values are reported for comparisons between the AUROC of fSENC- and FT-derived LV-GLS values.

To test their accuracy, the proposed cut-offs for fSENC- and FT-derived LV-GLS were applied to our study subgroups (*Figure 1*). Performance measures for all proposed cut-offs are summarized in *Table 2*.

At a threshold of −19.3%, fSENC-derived LV-GLS distinguished between healthy and HF with a sensitivity of 85.7% (95% CI 76.8%, 94.6%), a specificity of 68.4% (95% CI 47.4%, 89.5%), a positive predictive value of 88.9% (95% CI 82.4%, 95.7%), a negative predictive value of 61.9% (95% CI 45.8%, 80%), and an accuracy of 81.3% (95% CI 72%, 89.3%). The corresponding FT-derived LV-GLS threshold of −15.1% yielded a sensitivity of 85.7% (95 CI 76.7%, 94.6%), a specificity of 78.9% (95% CI 57.9%, 94.7%), a positive predictive value of 92.5% (95% CI 86.8%, 98%), a negative predictive value of 65.4% (95% CI 51.6%, 82.6%), and an accuracy of 84% (95% CI 76%, 92%) for the discrimination of healthy subjects and patients with HF.

Regarding the differentiation between HF with mild and severe DI, the fSENC-derived LV-GLS threshold of −15.8% resulted in a sensitivity of 88.9% (95% CI 72.2%, 100%), a specificity of 79% (95% CI 65.8%, 92.1%), a positive predictive value of 66.7% (95% CI 53.9%, 82.4%), a negative predictive value of 93.9% (95% CI 85.7%, 100%), and an accuracy of 82.1% (95% CI 71.4%, 91.1%). The corresponding FT-derived LV-GLS threshold of −10.8% yielded a sensitivity of 85% (95% CI 66.7%, 100%), a specificity of 73.7% (95% CI 57.9%, 86.8%), a positive predictive value of 60.7% (95% CI 46.7%, 76.2%), a negative predictive value of 90.6% (95% CI 80.8%, 100%), and an accuracy of 76.8% (95% CI 64.3%, 87.5%) for the discrimination of HF with mild and severe DI.

### Agreement between fSENC and FT

**Figure 3:**
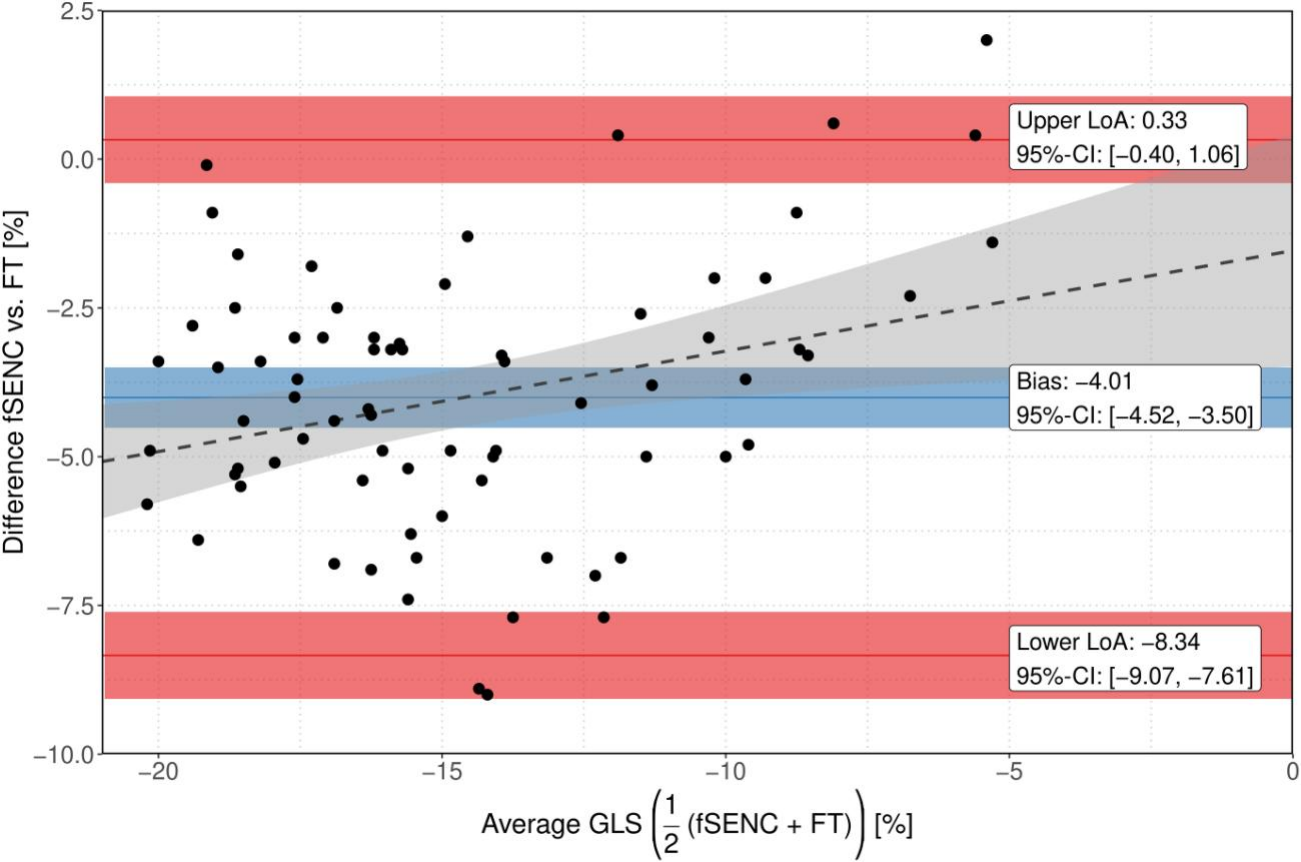
Bland–Altman plots for variability of fSENC-derived and FT-derived LV-GLS. The blue line indicates the mean difference (bias) between methods; the red lines, as indicated, show limits of agreement (95% CI of the differences of the measured values). Corresponding ribbons represent 95% CIs for the respective parameters. The grey dashed line reflects the increase of difference between the two methods for increasing LV-GLS values.

Bland–Altman analysis revealed that FT-derived LV-GLS values were generally higher than those derived by the reference method fSENC, with a bias of −4.01% (95% CI −4.52, −3.50) (*Figure 3*). The upper and lower limits of agreement were 0.33% (95% CI −0.25, 0.90) and −8.34% (95% CI −8.92, −7.76), respectively. A linear regression model for the difference between fSENC- and FT-derived LV-GLS given the mean LV-GLS acquired by the two methods revealed a significant positive association of 0.169 (95% CI 0.040, 0.298, *P* = 0.011), represented by the slope of the dashed grey line in *Figure 3*.

### Rescaling of LV-GLS values

**Figure 4:**
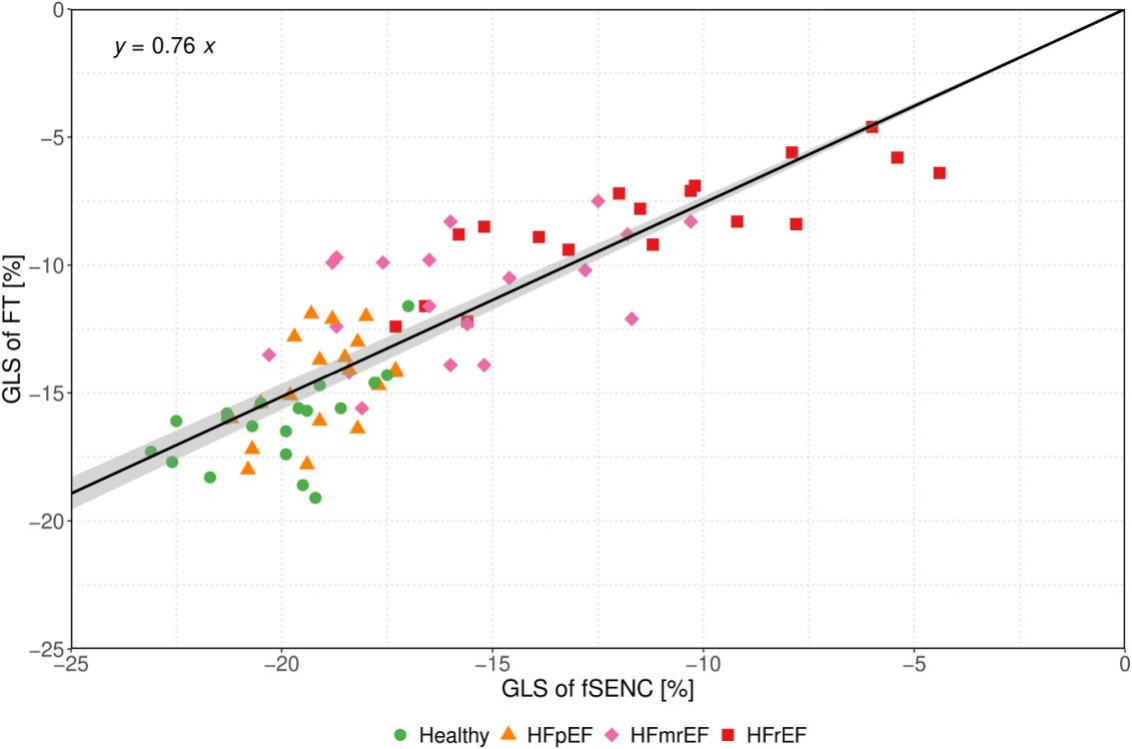
Linear regression model without intercept visualizing LV-GLS derived through FT vs. fSENC. The regression equation (*y* = 0.76*x*) is stated in the upper left corner. HF entities of individual patients are represented as different shapes and colours.

FT-derived LV-GLS values were plotted against those derived from fSENC using a linear regression model (*Figure 4*). As we expect the relationship between the FT-derived and fSENC-derived GLS to be a proportional one, we fitted a regression model without intercept. Of note, this model outperformed a linear regression model with intercept in terms of Akaike information criterion. In the intercept-free model, the slope was estimated as 0.76 (95% CI 0.732, 0.783). As suggested by the slope of this model, a factor of 0.76 was used to rescale the pre-defined fSENC-derived LV-GLS cut-offs, resulting in rescaled LV-GLS cut-offs for FT of −14.7% (−19.3% × 0.76) to discriminate between healthy subjects and HF with mild DI (i.e. HFpEF and HFmrEF) and of −12.0% (−15.8% × 0.76) to discriminate between HF with mild and severe DI (i.e. HFrEF) (*Table 2*).

### Accuracy of rescaled LV-GLS cut-offs

**Figure 5:**
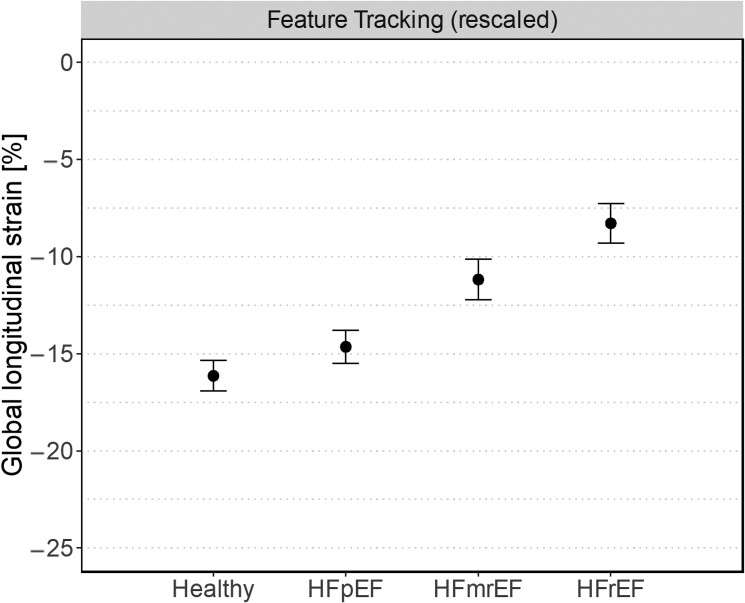
Scatter plot for FT-derived LV-GLS, stratified by entity of HF. Cut-off values are rescaled by a factor of 0.76 based on conversion from fSENC to FT. Within-group means are shown as point with corresponding 95% CI (error bar). Individual measurements are shown as small dots (jittered horizontally for better visibility). Rescaled FT LV-GLS cut-offs border the coloured regions. Specifically, rescaled LV-GLS cut-offs of −14.7% and −11.9% were used to discriminate between healthy subjects (green area) and those with HF (yellow and red area) and between HF with mild (yellow area) and severe DI (red area), respectively.

To assess their accuracy, we tested the rescaled FT LV-GLS cut-offs in our study subgroups (*Figure 5*). The rescaled FT LV-GLS cut-off of −14.7% discriminated between healthy subjects and patients with HF with a sensitivity of 83.9% (95% CI 75%, 92.9%), a specificity of 79% (95% CI 57.9%, 94.7%), a positive predictive value of 92.5% (95% CI 85.7%, 98%), a negative predictive value of 62.5% (95% CI 48.3%, 79%), and an accuracy of 82.7% (95% CI 73.3%, 90.7%). Equivalently the rescaled LV-GLS cut-off of −12.0% distinguished between HF with mild and severe DI with a sensitivity of 88.9% (95% CI 72.2%, 100%), a specificity of 65.8% (95% CI 50%, 79%), a positive predictive value of 55.2% (95% CI 45%, 68.2%), a negative predictive value of 92.9% (95% CI 82.8%, 100%), and an accuracy of 73.2% (95% CI 62.5%, 83.9%) (*Table 2*).

## Discussion

In the present study, we assessed differences in myocardial deformation between healthy subjects and different entities of HF (HFpEF vs. HFmrEF vs. HFrEF), using CMR LV-GLS derived by means of acquisition-based (i.e. fSENC) and post-processing (i.e. FT) techniques. The results of our study indicate that HF is associated with impaired myocardial deformation and show that LV-GLS is an effective tool to discriminate between healthy subjects and HF patients, as well as between HF with mild and severe DI, when well-defined cut-offs are used. LV-GLS values and, thus, the specific cut-off levels differ depending on the technique used to derive them. However, specific rescaling factors can be used to convert LV-GLS values and cut-offs derived by one technique to others.

While LVEF remains the standard variable by which HF is classified in clinical routine care, there is increasing evidence suggesting that myocardial strain analyses, whether performed via echocardiography or CMR, have superior diagnostic and prognostic value in patients with HF, mainly because they are more robust and provide more detailed information on global and regional myocardial contractility.^9–11^ Previous analyses from the present cohort have already shown that LV-GLS values are impaired in patients with HF compared with healthy controls and that LV-GLS values significantly deteriorate with increasing disease severity (i.e. HFpEF > HFmrEF > HFrEF), both findings that have since been reproduced in larger cohorts using CMR.^10,21,22^

We add to these analyses by demonstrating that LV-GLS, using specific cut-offs, can discriminate between healthy subjects and patients with HF, even in the setting of a preserved LVEF, and further discriminates between HF with mild (i.e. HFpEF and HFmrEF) and severe DI (i.e. HFrEF). This observation reinforces results from a large multi-centre study (*n* = 1230), which showed that various fSENC-derived myocardial strain indices, including LV-GLS, may be used to discriminate between healthy subjects, patients at risk, and patients with subclinical and clinical HF.^10^ Specifically, data from the above study demonstrated that at a cut-off of −19.5% LV-GLS discriminates between healthy subjects and those with subclinical HF with a sensitivity of 67% and specificity of 75%.^32^ This cut-off is well in line with the fSENC-derived LV-GLS cut-off (i.e. −19.3%) our data yielded for the discrimination between healthy subjects and HF with mild DI.^10^ In contrast, the LV-GLS cut-off proposed to discriminate between subclinical and clinical HF (i.e. −16.3%) was slightly lower than the one we identified to discriminate between HF with mild and severe DI (i.e. −15.8%). This may be explained by the fact that patients classified as having HF with mild DI in our study were already symptomatic and may have already had more severe myocardial contractility impairment.

Apart from those obtained in this study, no corresponding CMR-based cut-off values are currently available for FT-derived LV-GLS to the best of our knowledge. However, extensive data have previously demonstrated the ability of FT-derived LV-GLS to predict adverse cardiovascular events in both patients with and without HF.^33–35^ Unfortunately, several studies investigating the comparability of different techniques used to asses myocardial strain on CMR have shown that LV-GLS values vary significantly depending on the technique, vendor, or software used to assess them and that therefore cut-off values derived using one technique cannot simply be applied to those assessed using another technique.^36^ In line with these studies, we observed important discrepancies in mean LV-GLS measured by FT and fSENC. In particular, we observed a significant bias (−4.01%) towards higher (i.e. less negative) LV-GLS values when using FT compared with the reference method fSENC. Although the two methods showed good agreement in linear regression analysis (*Figure 4*), these results indicate that the quantitative results are not comparable. Such high variability makes it difficult to establish uniform reference values for LV-GLS and requires careful interpretation of results in clinical practice.

Hence, ours and other groups have previously emphasized the need for standardization and uniform interpretation of LV-GLS techniques.^15,37^ To address this issue, we herein used a linear regression model to obtain a straightforward mathematical method to convert LV-GLS between fSENC and FT. This approach suggested that a rescaling factor of 0.76 could be used to convert LV-GLS values and cut-offs derived using fSENC to FT with reasonable accuracy. Applying both FT-derived and rescaled FT LV-GLS cut-offs to our cohort, we observed a tendency of those based on dedicated FT-based measurements to discriminate healthy subjects, and HF patients with mild or severe DI with slightly higher accuracy than the rescaled FT LV-GLS cut-offs. Yet, the rescaled FT LV-GLS cut-offs still yielded accuracies of 82.7% and 73.2% for the discrimination between healthy and HF, as well as between HF with mild and severe DI, respectively. These results suggest that conversion using rescaling factors, such as the one we propose here, may be a valid approach to improve uniform interpretability of LV-GLS values measured using different techniques. Derivation and validation of rescaling factors allowing conversion of LV-GLS values between various acquisition techniques should be performed in larger cohorts before being applied in clinical routine care, though.

Comparison of the performance measures for fSENC- and FT-derived LV-GLS cut-offs, on the other hand, did not allow firm conclusions to be drawn. Specifically, FT-derived cut-offs tended to be more accurate for the differentiation between healthy subjects and patients with HF (accuracy 84% vs. 81.3%) and fSENC-derived cut-offs distinguished between HF with mild and severe DI with higher slightly higher accuracy (82.1% vs. 76.8%). However, the classification performance using fSENC- and FT-derived LV-GLS values did not differ significantly with respect to AUROC, neither for the discrimination between healthy subjects and HF patients [difference in AUROC −2.8% (95% CI −9.3%, 3.8%, *P* = 0.406)] nor between HF with mild and severe DI [difference in AUROC 0.6% (95% CI −4.8%, 6%, *P* = 0.831)], indicating that the overall performance of both techniques was comparable.

**Figure 6:**
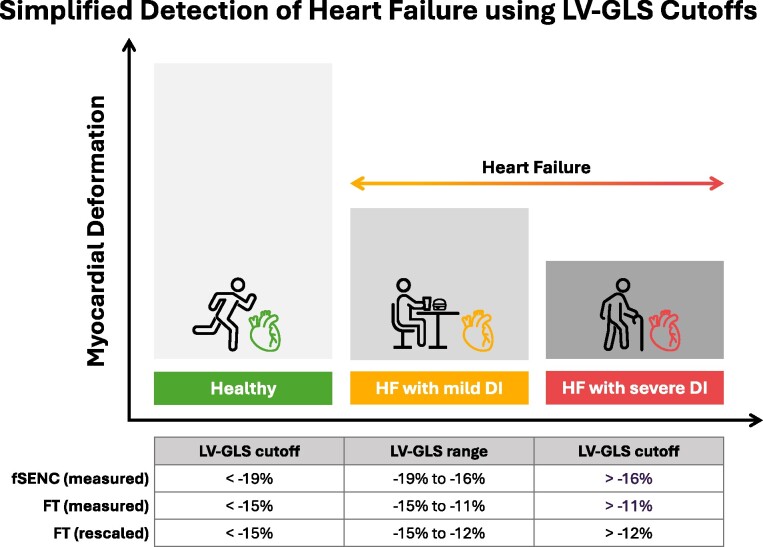
Simplified approach for the identification of HF using fSENC-based, FT-based, and rescaled FT LV-GLS in clinical routine care. Using the cut-offs summarized in the table, the proposed approach can be used to discriminate between healthy subjects, HF with mild DI, and HF with severe DI.

Taken together, our results demonstrate that LV-GLS values can be used to discriminate between healthy subjects and patients with HF and to further classify those with HF into groups with mild and severe DI, irrespective of the technique (i.e. fSENC vs. FT) used to quantify them. On the basis of this information and the fundamental advantage that FT does not require acquisition of dedicated CMR sequences, allowing for uniform application on routinely acquired images, we propose a simplified FT-based approach for the early detection of HF in clinical routine care, in which an LV-GLS < −15% identifies healthy subjects and an LV-GLS > −15% indicates HF. In addition, HF with mild DI can be distinguished from HF with severe DI (*Figure 6*). Numerically, our proposed approach is in good agreement with extensive echocardiographic data, including a recent meta-analysis based on 44 studies and a total of 8910 patients, which indicated that LV-GLS values below a threshold of −14.7% should be considered abnormal.^38,39^

## Limitations

Although our study cohort remains well characterized, the main limitation of our analyses is the relatively small sample size. Furthermore, it must be acknowledged that, when performing GLS measurements, differences in post-processing or image acquisition such as slice inclusion or planning, will affect the strain values obtained. Therefore, our proposed conversion may not be applicable to FT-derived values obtained with other software packages. This scenario may require additional adaptation.

Finally, in this study, we only compared two of several techniques to measure myocardial strain in this study, excluding, for example, conventional myocardial tagging, for instance, which is considered as the gold standard for strain measurements.^12,13^ Previous studies, however, have validated both fSENC and FT against myocardial tagging.^12,13^

## Conclusions

LV-GLS accurately discriminates between healthy subjects and those with HF. Due to differences in quantitative output, specific cut-offs are required when using different measurement techniques. However, rescaling factors may facilitate conversion between different techniques, potentially facilitating uniform application of cut-offs in clinical practice. FT-based LV-GLS values ≥ −15% may be used as a simplified approach to identify HF in clinical routine care.

## Supplementary data

Supplementary data are available at *European Heart Journal - Imaging Methods and Practice* online.

## Supplementary Material

qyae093_Supplementary_Data

## Data Availability

The data underlying this analysis will be shared on reasonable request to the corresponding author.

## Lead author biography

Dr Undine Witt graduated in medicine from the Technical University of Dresden (Germany) and completed her specialist cardiologist training at the Department of Cardiology at Charité University Medicine in Berlin. Afterwards, she worked at the German Heart Centre Berlin as a study doctor in a large CMR project about early detection of HF patients and also in clinical CMR as part of the MRI core lab of Prof. Sebastian Kelle. She currently works in a cardiological practice.
