# Supplementary material for: A simplified approach to discriminate between healthy subjects and patients with heart failure using cardiac magnetic resonance myocardial deformation imaging
Source: Eur Heart J Imaging Methods Pract. 2024 Sep 12;2(3):qyae093. doi: 10.1093/ehjimp/qyae093 (PMC11421468; doi:10.1093/ehjimp/qyae093)
Supplement: qyae093_Supplementary_Data [file qyae093_supplementary_data.docx]

Supplementary Material

**Supplementary Material S1:**


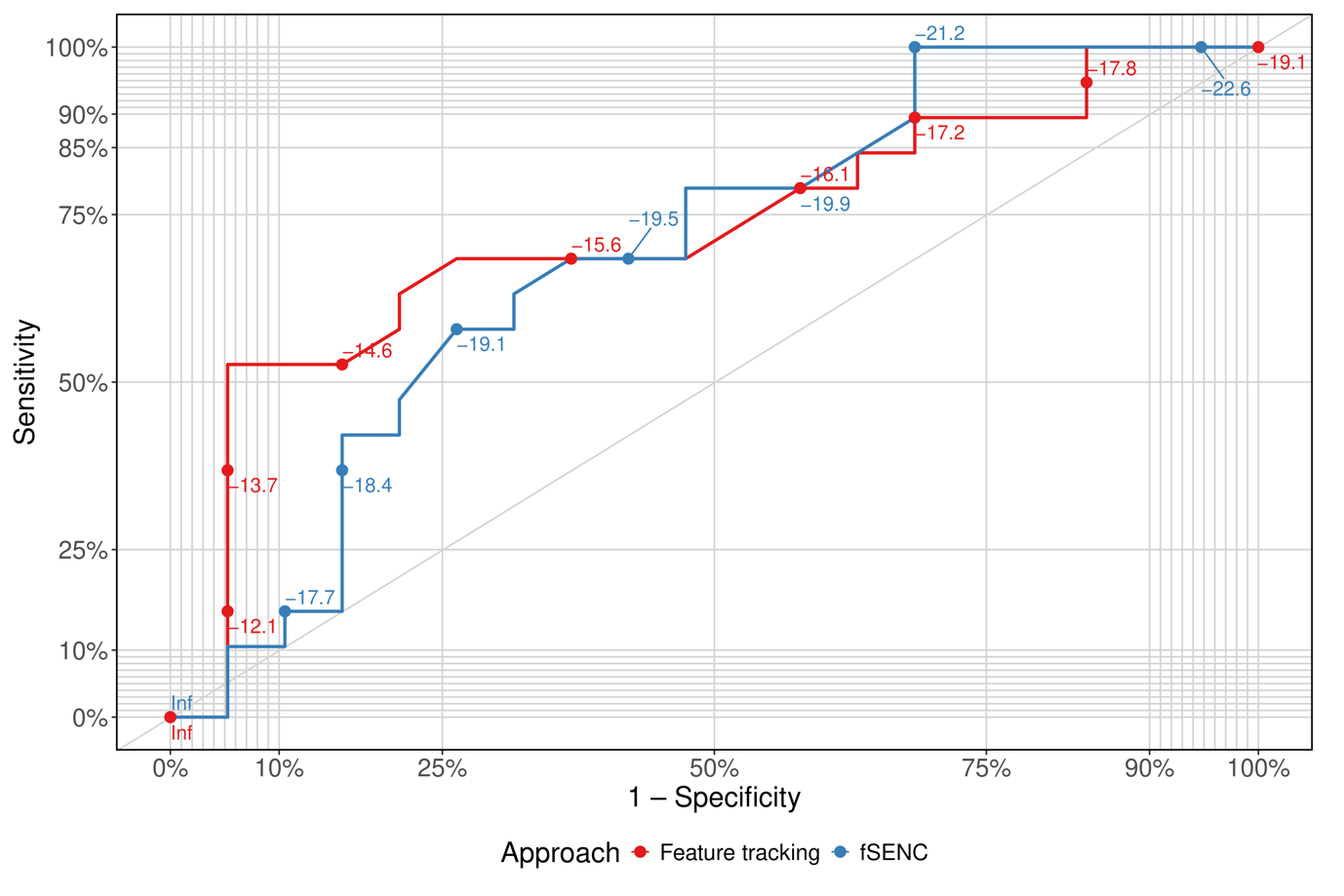


**Supplementary Material S1:** Receiver operating characteristics (ROC) curves of the comparison of feature tracking (FT)-based (red) and fast strain-encoded imaging (fSENC)-based (blue) LV-GLS for the discrimination between healthy subjects and patients with heart failure and preserved ejection fraction (HFpEF).

**Supplementary Material S2:**

|  | **Healthy vs. HFpEF** | |
| --- | --- | --- |
| **Performance parameter** | **fSENC** | **FT** |
| AUROC | 68.8% [51.4%; 86.3%] | 72.6% [55.7%; 89.5%] |
| Comparison of method‘s AUROC vs. random classifier | p = 0.049 | p = 0.018 |
| Comparison between AUROC of fSENC and FT | p = 0.634 | |

**Supplementary Material S2:** Areas under the receiver operating characteristic curve (AUROC) regarding the differentiation between healthy subjects and patients with heart failure and preserved ejection fraction (HFpEF) for continuous fast strain-encoded imaging (fSENC)-derived and feature tracking (FT)-derived left ventricular global longitudinal strain (LV-GLS). P-values are given for comparing each AUROC with a random classifier as well as for the comparison between AUROCs of fSENC- and FT-derived LV-GLS values.
